# Institutional learning curve and factors of prolonged operation time of robotic distal pancreatectomy: An analysis of an initial 117 cases

**DOI:** 10.1002/ags3.70005

**Published:** 2025-02-24

**Authors:** Yuki Hirata, Laura Prakash, Jess Maxwell, Rebecca Snyder, Michael Kim, Hop Tran Cao, Ching‐Wei D. Tzeng, Jefferey E. Lee, Matthew H. G. Katz, Naruhiko Ikoma

**Affiliations:** ^1^ Department of Surgery National Hospital Organization Tokyo Medical Center Tokyo Japan; ^2^ Department of Surgical Oncology The University of Texas MD Anderson Cancer Center Houston Texas USA

**Keywords:** learning curve, pancreatectomy, robotic pancreatectomy

## Abstract

**Background:**

The granular methods by which centers can safely implement and effectively expand robotic distal pancreatectomy (RDP), including those related to appropriate patient selection during the learning curve period, remain unclear. This study aimed to verify that our strategic robotic surgical oncology program effectively implemented RDP as standard practice and to identify factors associated with prolonged operation time.

**Materials and Methods:**

We performed a detailed analysis of the intraoperative and short‐term outcomes of consecutive patients (October 2018–September 2023) undergoing RDP at our center, beginning with the first patient in our program. Operation time was analyzed using a cumulative sum chart (CUSUM), and factors associated with prolonged operation time were analyzed.

**Results:**

Throughout the study period, five surgeons performed RDP for 117 patients. The CUSUM analysis indicated our center required 18 cases to overcome the initial learning phase and 43 additional cases to become proficient. In contrast, when comparing short‐term outcomes across the three observation periods, there were no significant changes in the incidence of ACCORDION grade ≥3 pancreatic fistulas (*p* = 0.684), or readmission rates (*p* = 0.457). A multivariable analysis revealed BMI ≥30 in male, the presence of pancreatitis or fibrosis, and the performance of concomitant procedures were associated with extended operation times, while BMI ≥30 in female was not.

**Conclusions:**

Although an institutional learning curve was observed, our program enabled the safe implementation of RDP and successfully expanded the number of primary operating surgeons while maintaining stable short‐term outcomes. The absence of an impact of high BMI on operation time in female patients suggests a notable advantage of robotic approach for these individuals.

## Graphical abstract

This study verified the effective implementation of robotic distal pancreatectomy as the standard approach in our strategic robotic surgical oncology program. Although an institutional learning curve was observed, the robotic surgery program successfully expanded the number of primary operating surgeons while maintaining stable short‐term outcomes.

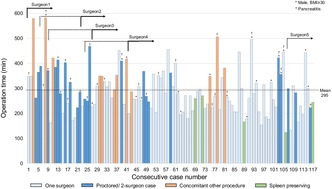

## 1 INTRODUCTION

The use of minimally invasive approaches, particularly those that entail robotic surgery methods, has sharply increased for cancer‐related operations, including for pancreatectomy.^1^, ^2^ The surgical safety as well as oncological quality of robotic pancreatectomy is still under investigation, particularly those for pancreatoduodenectomy,^3^ but there is accumulating evidence supporting the use of minimally invasive surgery and a robotic approach for distal pancreatectomy (RDP).^4^ Compared to an open approach, laparoscopic distal pancreatectomy (LDP) results in less blood loss, fewer incidences of delayed gastric emptying, and shorter time to functional recovery.^5^ Compared to a laparoscopic approach, RDP has reduced rates of conversion to open laparotomy, increased the number of lymph nodes examined, and improved negative resection margin rates.^6^, ^7^ However, the studies that evaluated robotic approach were conducted in high‐volume institutions for minimally invasive pancreatectomy (e.g., over 1000 pancreatectomies performed annually at an institute and each surgeon had to experience at least 50 laparoscopic pancreatectomies before RDP training).^8^ Previous reports found that surgeons require 10 to 67 cases to become proficient at RDP,^9^, ^10^, ^11^, ^12^ with higher incidences of postoperative pancreatic fistula, higher readmission rates, fewer number of lymph nodes examined, as well as prolonged operation time during such leaning curve periods.^9^ The incidence of postoperative complications is associated with impaired oncological outcomes.^13^ The oncological qualities of an operation, such as number of lymph nodes examined and negative margin resection, are associated with survival.^14^, ^15^, ^16^ Thus, it is critical for centers to carefully implement a robotic surgery program without compromising surgical and oncological safety, enabling patients to enjoy the potential benefits of minimally invasive RDP even during the learning‐curve period. Training national and international programs for robotic pancreatectomy exist, championed by surgeons from the University of Pittsburg Medical Center.^17^, ^18^ However, access to such established programs is limited. In 2018, our institution strategically initiated a robotic surgical oncology program with prospective safety monitoring.^19^ Our program performs various types of upper gastrointestinal cancer operations, including pancreatectomy, hepatectomy, and gastrectomy, effectively increasing the composite case volume and shortening the learning curve for individual surgeons. We increased the case complexity and the number of surgeons over time to safely expand the program.^20^

Overall, it is unknown how centers can safely implement and effectively expand a robotic surgery program, particularly at a cancer center, which lacks non‐malignant procedures often used by surgeons to gain robotic skills. Moreover, it is unknown what factors are associated with prolonged operation time in RDP, making it difficult to guide appropriate patient selection during the learning‐curve period. The objectives of this study are to confirm the effective implementation of RDP as a standard practice under our program and identify factors associated with prolonged operation time. Thus, we analyzed our initial 5‐year experience of RDP to investigate changes in operation time over time as a surrogate of operative efficiency to define our learning curve using cumulative sum chart (CUSUM) analyses.

## 2 MATERIALS AND METHODS

### 2.1 Study design and patients

After obtaining approval from our institutional review board, we retrospectively queried a prospectively maintained departmental database for pancreatic surgery to collect clinicopathologic and operation data for patients who underwent RDP from October 1, 2018, through September 30, 2023, at The University of Texas MD Anderson Cancer Center. At our institution, treatment and surgical strategies are discussed at multidisciplinary meetings as well as preoperative surgical meetings for individual patients. This study was approved by the institutional review board (PA18‐0854).

### 2.2 RDP in our institute

To perform RDPs, we used the Da Vinci Xi Surgical System (Intuitive Surgical, Inc., Sunnyvale, CA, USA) for all but one patient. Our strategies for establishing a robotic surgical oncology program involved synergistic learning of robotic skills across a range of oncological procedures, bolstered by a higher composite case volume. We also concentrated cases to a program lead‐surgeon (surgeon 1) to expedite the learning curve, followed by a phased integration of other surgeons into the program. Our surgical quality committee requires that a surgeon complete at least five robotic operations while being proctored before performing robotic operations independently. Beyond these five proctored cases, surgeon 1 continued to serve as a co‐surgeon when other surgeons performed RDP when requested. Surgeons were not required to have previous laparoscopic experience to be included in the program. Additionally, surgeons who performed RDP had been trained in surgical oncology and had robust prior experience with open pancreatectomy.

### 2.3 Variables

We collected the following patient and tumor characteristics from the prospective database: age, sex, race or ethnicity, body mass index (BMI), histological diagnosis of the tumor, and use and types of neoadjuvant therapy. Surgical variables included operating surgeon(s), operation time (from incision to closure of all incisions), estimated blood loss, conversion to open laparotomy, procedure types (distal pancreatectomy [DP] with splenectomy, spleen‐preserving DP, and DP with splenectomy and other concomitant procedures), site of pancreatic transection (neck or body), surgeon‐reported intraoperative findings of pancreatitis and/or fibrosis (moderate or severe that affected the difficulty of the operation), length of hospital stay, incidence and severity of postoperative complications (classified using the ACCORDION severity grading system^21^) including pancreatic fistula, delayed gastric emptying, and postoperative readmission within 90 days of surgery. The prospective database was reviewed biweekly with one surgeon and at least two advanced practice providers.^22^

### 2.4 Statistical analyses and institutional learning curve analyses

We included consecutive patients who underwent RDP during the study period, starting with the first patient of our program. First, we summarized the trend of operation time and characteristics of procedures (single‐ or two‐surgeon cases and details of the procedure type), as well as the operational timing for each surgeon when they were proctored and independent. Then, to define institutional and individual learning curves, we depicted the transition of operation time using the CUSUM analysis for all cases and for surgeon 1's individual cases. The CUSUM represents the cumulative difference between each operation time data point and the overall mean operation time, starting from the first case and after adding each subsequent case. We categorized the analysis period into three phases based on the inflection points of the curve: the initial learning phase; the increased competence phase; and the mastery phase.^23^ Additionally, under the assumption that the presence of a learning curve was uncertain, we simply divided the 117 consecutive cases into three equal parts (the first 40 cases (1–40); the second 40 cases (41–80); and the last 37 cases (81–117)). Postoperative short‐term outcomes (any complications ≥ grade 2, pancreatic fistula ≥ grade 3, and the incidence of readmission within 90 days) were then compared using one‐way ANOVA. Based on these analyses, we comprehensively examined the institution‐wide learning curve and evaluated the surgical safety of our RDP program during this implementation period.

To identify factors associated with longer operation times, linear regression models were fitted. Factors with *p*‐values <0.20 in the univariable analysis were included in the multivariable model. To examine whether the effect of BMI differs by sex, we created a variable with categories based on sex and BMI with a cutoff of 30, which is commonly used to define obesity.^24^ All statistical tests were two‐sided, and *p*‐values <0.05 were considered statistically significant. All statistical analyses were conducted using Stata software version 14.1 (StataCorp, College Station, TX, USA).

## 3 RESULTS

### 3.1 Patient, surgical, and pathological characteristics

We identified and included 117 patients (Table 1). The median age was 63 years and 52% were male and 68% were non‐Hispanic White. The median BMI was 29 (range 19–73). Histologic findings included neuroendocrine tumor (38%), cystic disease (37%), and adenocarcinoma (17%). Nineteen patients (16%) underwent neoadjuvant therapy before surgery, and 100 patients (85%) underwent DP with splenectomy, five patients (4%) underwent spleen‐preserving DP, and 12 patients (10%) underwent DP with splenectomy and concomitant other procedures. Surgeons reported the presence of pancreatitis and/or fibrosis that increased the difficulty of surgery in 19 patients (16%). The median operation time was 279 minutes, and estimated blood loss was 50 mL. Seventy percent of cases were performed by a single surgeon and 30% by two surgeons, which included planned proctor cases and cases with an intraoperative request for a co‐surgeon. No patients required conversion to open laparotomy. The pancreas was transected at the neck in 56 patients (48%).

**TABLE 1:** Clinical, operative, and pathologic characteristics (*N* = 117).

	*n* (%)
Age (years)^a^	63 (16–83)
Sex	
Male	61 (52)
Female	56 (48)
Race/ethnicity	
Non‐Hispanic White	79 (68)
Non‐Hispanic Black	7 (6)
Asian	8 (7)
Hispanic/Latino	19 (16)
Other	4 (3)
BMI (kg/m^2^)^a^	29 (19–73)
Histology diagnosis	
Adenocarcinoma	20 (17)
Cystic disease	43 (37)
Neuroendocrine tumor	44 (38)
Primary pancreatic tumor, other	5 (4)
Metastatic pancreatic tumor	5 (4)
Neoadjuvant chemotherapy	19 (16)
Site of pancreatic transection	
Neck	56 (48)
Body	61 (52)
Procedure type	
DP with splenectomy	100 (85)
Spleen‐preserving DP	5 (4)
DP with splenectomy + other concomitant procedures^b^	12 (10)
DP with splenectomy + cholecystectomy	4
DP with splenectomy + ventral hernia repair	2
DP with splenectomy + adrenalectomy	3
DP with splenectomy + microwave ablation (liver)	1
DP with splenectomy + gastrectomy	1
DP with splenectomy + hepatectomy	4
Pancreatitis, fibrosis (moderate or higher)	19 (16)
Operation time (min)^a^	279 (124–582)
Estimated blood loss (mL)^a^	50 (5–800)
Converted to open laparotomy	0 (0)
Site of pancreatic transection	
Neck	56 (48)
Body	61 (52)
Length of hospital stay, (days)^a^	3 (2–7)
Postoperative complications	
Any complications ≥ grade 2	37 (32)
Delayed gastric emptying ≥ grade 2	1 (1)
Pancreatic fistula ≥ grade 3	11 (9)
Readmission within 90 days after surgery	17 (15)

*Note*: Complication grades are according to the ACCORDION classification.

Abbreviations: BMI, body mass index; DP, distal pancreatectomy.

^a^ Median (range).

^b^ There are overlapping.

### 3.2 Expansion of RDP program and trend of operative time

**FIGURE 1:**
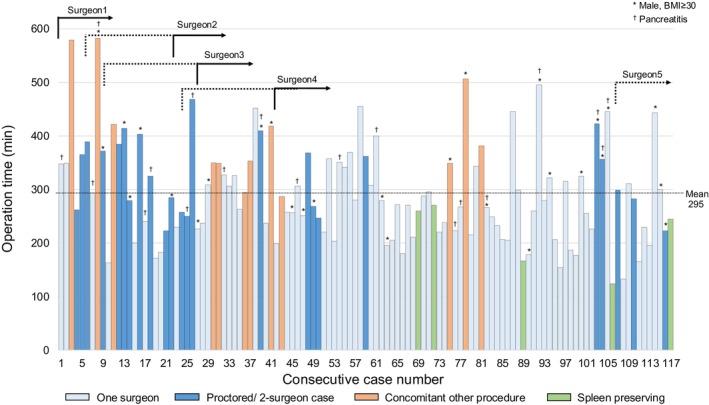
Operation time of all robotic distal pancreatectomy cases, type of procedure, and the timing when the surgeons began their training (dash lines) and when they became the primary operating surgeons (solid lines). For concomitant cases involving two surgeons, we prioritized classifying them as concomitant (orange). All spleen‐preserving distal pancreatectomy cases (green) were performed by a single surgeon.

The annual case volume increased over time: 19 cases in 2018–2019, 17 cases in 2019–2020, 25 cases in 2020–2021, 29 cases in 2021–2022, and 31 cases in 2022–2023. During this study period, five surgeons performed RDP. The trend of operation time and the timing of each surgeon starting training and becoming the primary operating surgeon are visualized on Figure 1. Notably, a high proportion (52%) of the first 50 cases were performed by two surgeons (all two‐surgeon cases included surgeon 1), while cases were more often (78%) performed by one surgeon in the later study period (after 50 cases). Most of the prolonged cases had concomitant procedures, male patient with BMI ≥30, and/or moderate/severe pancreatitis. Overall, we observed a gradual trend of shorter operative time as our program gained experience.

### 3.3 Learning curve analyses

**FIGURE 2:**
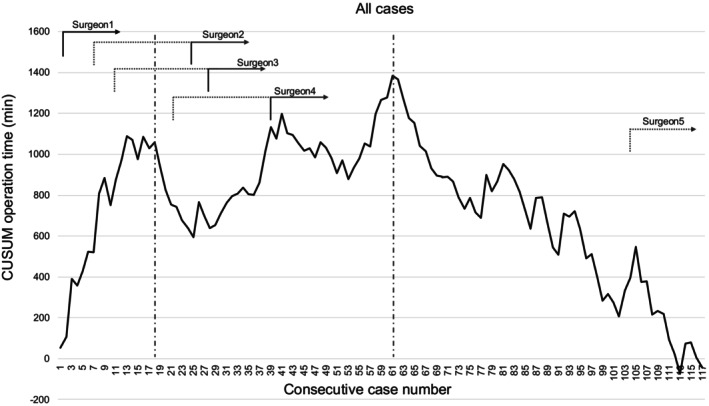
The cumulative sum chart (CUSUM) curve of all cases' operation times (institutional). Dash and solid lines, respectively, indicate whether the surgeon was in training or the primary surgeon.

**FIGURE 3:**
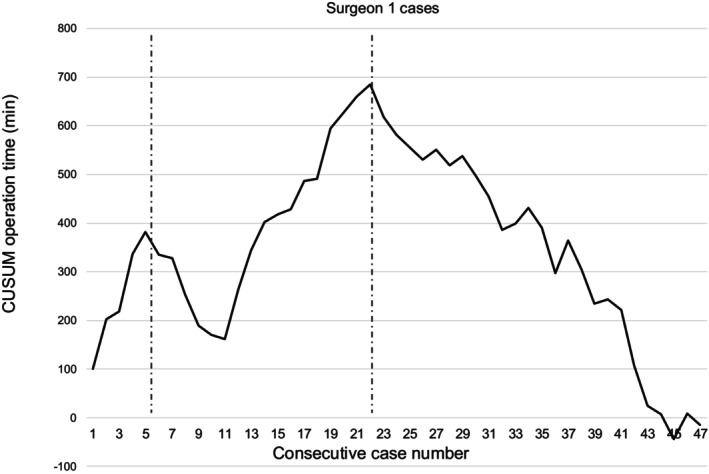
The CUSUM curve of surgeon 1's operation times (individual).

CUSUM analyses based on operation time were conducted (Figure 2). Based on this, we identified three phases of the institutional learning curve: the first phase from the 1st to the 18th case (i.e., the initial learning phase), the second phase from the 19th to the 61st case (i.e., the increased competence phase), and the third phase from the 62nd to the 117th case (i.e., the mastery phase). The CUSUM analysis of individual learning curve analysis using cases performed by surgeon 1 (Figure 3) showed lower numbers needed in each phase, demonstrating differences between institutional and individual learning curves: the first phase 1st–5th case, the second 6th–22nd case, and the third 23rd–47th case.

Analyses of short‐term outcomes across three time periods (Table 2) showed consistently low incidences of complication and readmission rates during the study period. This indicated the safe implementation of our RDP program, both during the initial period and as the number of surgeons performing RDP increased.

**TABLE 2:** Complications and readmission by time periods, *n* (%).

Case number	1–40	41–80	81–117	*p‐*value
Any complication ≥ grade 2	11 (28)	14 (35)	12 (32)	0.765
Pancreatic fistula ≥ grade 3	4 (10)	3 (8)	5 (14)	0.684
Readmission	8 (20)	4 (10)	6 (16)	0.457

### 3.4 The factors associated with longer operation time

In the multivariable analyses (Table 3), cases 60 and above (i.e., cases during the mastery phase) were associated with shorter operative time (coefficient [in minutes] = −48.1, 95% confidence interval [CI] = −88.0 to −8.2, *p* = 0.018). BMI ≥30 in male patients was associated with longer operative time by more than 1 hour (coefficient = 82.7, 95% CI = 29.5 to 135.9, *p* = 0.003), while BMI ≥30 in female patients was not (coefficient = −0.15, 95% CI = −46.7 to 46.4, *p* = 0.995). The presence of pancreatitis or fibrosis (coefficient = 47.5, 95% CI = 7.1 to 87.8, *p* = 0.026) and undergoing concomitant procedures (coefficient = 102.6, 95% CI = 57.0 to 148.2, *p* < 0.001) were also strongly associated with longer operation time. Conversely, pancreatic transection at the body (vs. at the neck) was associated with shorter operation times (coefficient = −50.4, 95% CI = −80.1 to −20.7, *p* = 0.001).

**TABLE 3:** Association between operation time and variables.

Variable	Coefficient	95% confidence interval	*p*‐value
*Univariable analysis*			
Age (ref. <65 years)			
≥65 years	11.2	−22.5 to 44.9	0.512
Sex and BMI (ref. female, BMI <30)			
Male, BMI <30	10.7	−25.8 to 47.1	0.563
Male, BMI ≥30	107.8	49.3 to 166.6	**<0.001**
Female, BMI ≥30	2.8	−49.5 to 55.0	0.916
Histological type (ref. adenocarcinoma)			
Cystic	17.1	−32.8 to 67.0	0.498
Other	8.1	−39.2 to 55.5	0.735
Pancreatic transection at body (ref. neck)	−44.0	−76.7 to −11.4	**0.009**
Pancreatitis or fibrosis	73.9	30.4 to 117.4	**0.001**
Institution's experience (ref. 1–20 cases)			
21–40 cases (initial learning phase)	−28.7	−84.6 to 27.2	0.311
41–60 cases (increased competence phase)	−31.2	−87.0 to −24.7	0.272
After 61 cases (mastery phase)	−64.5	−110.4 to −18.5	**0.006**
Procedure type (ref. DP with splenectomy)			
Spleen preserved	−49.3	−127.7 to 29.2	0.216
Concomitant procedure	93.9	43.5 to 144.4	**<0.001**
*Multivariable analysis*			
Age (reg. <65)			
≥65	3.2	−25.8 to 32.2	0.648
Sex and BMI (ref. female, BMI <30)			
Male, BMI <30	18.9	−14.7 to 52.6	0.266
Male, BMI ≥30	82.7	29.5 to 135.9	**0.003**
Female, BMI ≥30	−0.15	−46.7 to 46.4	0.995
Pancreatic transection at body (ref. neck)	−50.4	−80.1 to −20.7	**0.001**
Pancreatitis or fibrosis	47.5	7.1 to 87.8	**0.026**
Institutional experience (ref. 0–20)			
21–40 (initial learning phase)	−23.8	−71.8 to 24.3	0.487
41–60 (increased competence phase)	−18.9	−67.1 to 29.2	0.076
After 61 (mastery phase)	−48.1	−88.0 to −8.2	**0.018**
Procedure type (ref. DP with splenectomy)			
Spleen preserved	−17.3	−89.7 to 55.1	0.637
Concomitant other procedure	102.6	57.0 to 148.2	**<0.001**

Abbreviations: BMI, body mass index; DP, distal pancreatomy.

*Note*: Bold values indicate statistical significance (*p* < 0.05).

## 4 DISCUSSION

In this retrospective cohort study, we conducted a detailed analysis of the surgical and short‐term outcomes of 117 consecutive patients who underwent RDP at our cancer center during the learning‐curve period and evaluated the implementation and dissemination of our robotic surgery program for safety and effectiveness, respectively. To maximize our early experience, surgeons frequently performed RDP collaboratively, reflecting our strategic and collegial team commitment to build a robust robotic surgery program with prospective safety monitoring. Our strategic model allowed a lead surgeon to overcome the initial learning phase quickly (*n* = 5), establish our institution's standard of operative details for RDP and safely disseminate robotic surgery expertise to other surgeons without compromising surgical safety. Notably, we have not experienced a case needing conversion to laparotomy. Short‐term outcomes of RDP were satisfactory relative to the benchmark cutoffs set by the most recent experts' consensus^25^ as well as our historical institutional data^26^ and remained stable from our very early experience as we improved efficiency by reducing operative time as our expertise matures. Overall, this study confirmed that the implementation of our institutional robotic RDP program was safe and effective and would provide valuable insights for institutions that are going to implement robotic surgical oncology programs.

When implementing novel surgical techniques, a learning curve is unavoidable. Typically, the learning curve for surgery is assessed using the CUSUM of operation time.^23^, ^27^, ^28^ However, operation time is influenced by a variety of factors such as the surgeon's prior experience, frequency of operations, and patient‐related factors. The number of surgeons who are included in the analysis affects number of cases needed to overcome learning curve (i.e., the more surgeons included, the more cases needed to achieve the learning curve as shown in our results). It is also known that when using CUSUM, a higher number of cases included in an analysis result in an elevated number of cases required to overcome the learning curve.^23^ More importantly, operating time is only one aspect of determining the learning curve, and it is critical that the institution overcomes the learning curve without increasing complications when implementing a robotic surgery program and when including new surgeons. From this viewpoint, our model, characterized by a collegial working environment, was deemed successful. Our program is likely sustainable and expandable with the addition of more new surgeons, and our model holds the potential for reproduction at other cancer centers as well.

We developed a robotic pancreatectomy program under circumstances where open pancreatectomy was the standard of care with relatively limited prior experience in laparoscopic pancreatectomy. The transferability of experience from laparoscopic to robotic surgery remains a controversial topic.^29^ We consider the skills required for robotic surgery to be fundamentally different from those for laparoscopic surgery, other than basic knowledge of minimally invasive techniques. Thus, in complex operations such as pancreatectomy, surgeons must overcome the learning curves of each surgical approach, including open, laparoscopic, and robotic.^8^ Indeed, the ROLARR randomized clinical trial^30^ demonstrated that the potential benefits of robotic surgery were observed when performed by surgeons with substantial prior experience in robotic proctectomy. However, it showed that the level of laparoscopic experience was not associated with outcomes in robotic proctectomy.^30^ A similar concept can be applied to proficiency in a robotic pancreatectomy program, a conclusion that is supported by the safe implementation of our robotic pancreatectomy program.

We used operative time as a parameter of surgeons' proficiency to define our learning curve. Shorter operation time reduces medical cost and labor cost for efficient use of resources.^31^ Appropriate patient selection during early experience is important, but there are scarce data to guide such patient selection. Our study identified intraoperative findings of pancreatitis or fibrosis, pancreatic transection at neck (vs. body), and concomitant other procedures were associated with longer operation time. Notably, a BMI ≥30 substantially lengthened the operative time by more than 1 hour in male patients but not in female patients. Obesity is known to be a risk factor for intraoperative difficulty in abdominal surgery,^32^, ^33^ and prior studies have shown a higher rate of complications among obese patients undergoing minimally invasive pancreatectomy.^34^ The use of a robotic approach has been reported to mitigate the effects of patients' obesity compared to a laparoscopic approach.^35^ However, our results suggest that for facilities implementing RDP, preferable patient selection criteria include female patients regardless of BMI, male patients with BMI <30, tumor lesions that can be resected at the pancreatic body (without a need for pancreatic tunneling at the neck), and patients without predictive signs of pancreatitis or fibrosis.

This study has several potential limitations. First, this study was conducted in a comprehensive cancer center in the United States, which could limit this study's external validity to more general surgical centers while establishing a model potentially reproducible in specialized surgical centers. We used CUSUM to analyze the learning curve and visually identified each learning phase. There are known limitations in CUSUM analyses, one of which is the subjective nature of defining learning phases. Additionally, the learning phase tends to become longer as more cases are added to the CUSUM analysis. From this perspective, it is likely that we have underestimated the duration of the learning phase. The heterogeneity of the procedures, which included concomitant procedures and co‐surgeon cases, made interpretation of CUSUM analyses somewhat complicated, but excluding those cases skews the study results. Thus, we included such cases in our analyses. Learning phase analyses for an institution or surgeon are also prone to errors in interpretation. We considered separating learning phase analyses for each surgeon, but in our collegial environment, this was deemed neither feasible nor valid. In two‐surgeon cases, the level of involvement of surgeon 1 varied depending on the proficiency of the other surgeon. Even after a surgeon was considered “independent,” we continued to support each other whenever needed. That said, this collegial approach should be maintained to ensure the safety and success of expanding the robotic surgical oncology program. Simultaneous experience with other robotic procedures, such as gastrectomy and pancreatoduodenectomy, during this study period likely enhanced the learning experience of surgeons but may have led to an underestimated learning curve for RDP. The terms used in this study may also have certain limitations. We applied the ACCORDION grading system for postoperative complication classification, as it aligns with our prospective management database, ensuring consistency with our previous reports.^22^ We did not use pancreatic texture in our analysis because we believe its impact on the difficulty of surgery in DP is limited. Instead, we consider the presence of pancreatitis and fibrosis, as used in this study, to be more significant contributors to surgical difficulty in DP. Despite these limitations, our granular analysis, which included all cases from the initial introduction without exclusion, is unique and a strength of this study.

In conclusion, while an institutional learning curve was observed in operation time, our robotic surgery program with a surgeon‐mentor model enabled the safe implementation of RDP and successfully expanded the number of primary operating surgeons with stable short‐term outcomes. Factors such as male obesity (BMI ≥30), pancreatitis or fibrosis, and the necessity for concomitant procedures played a significant role in prolonging operation time. Our results provide valuable insights for the institutions that plan to implement robotic surgical oncology programs.

## AUTHOR CONTRIBUTIONS

**Yuki Hirata:** Conceptualization; data curation; formal analysis; writing – original draft. **Laura Prakash:** Data curation; writing – review and editing. **Jess Maxwell:** Data curation; writing – review and editing. **Rebecca Snyder:** Data curation; writing – review and editing. **Michael Kim:** Data curation; writing – review and editing. **Hop Tran Cao:** Data curation; writing – review and editing. **Ching‐Wei D. Tzeng:** Data curation; writing – review and editing. **Jefferey E. Lee:** Data curation; writing – review and editing. **Matthew H. G. Katz:** Data curation; supervision; writing – review and editing. **Naruhiko Ikoma:** Conceptualization; data curation; formal analysis; supervision; writing – original draft; writing – review and editing.

## FUNDING INFORMATION

No funding was received for conducting this study.

## CONFLICT OF INTEREST STATEMENT

N.I. received a clinical research grant from Intuitive Surgical in 2021, but this funding did not directly support this study. N.I. is an editorial board member of *AGS*. All other authors involved in this study have disclosed any potential conflicts of interest and no conflicts were identified. The authors have not received any financial support that could be interpreted as having influenced the study design, data collection, analysis, interpretation, or the decision to publish.

## ETHICS STATEMENT

Approval of the research protocol: This study was approved by the MD Anderson Institutional. Review Board (protocol number PA18‐0854).

Informed Consent: N/A.

Registry and the Registration No. of the study/Trial: N/A.

Animal Studies: N/A.
